# The effect of toll-like receptor 7/8 ligand in inhibiting the motility of putative X-chromosome-bearing sperm in rams

**DOI:** 10.5455/javar.2024.k814

**Published:** 2024-09-29

**Authors:** Rangga Setiawan, Rini Widyastuti, An An Nurmeidiansyah, Nurcholidah Solihati

**Affiliations:** Department of Animal Production, Faculty of Animal Husbandry, Universitas Padjadjaran, Bandung, West Java, Indonesia

**Keywords:** Sexed sperm, toll-like receptor 7/8 (TLR7/8), motility, ram

## Abstract

**Objectives:**

This study aims to determine the effect of a toll-like receptor 7/8 (TLR7/8) ligand on the motility of putative X- and Y-chromosome-bearing sperm in rams.

**Materials and Methods:**

Sperm from three fertile rams were incubated with tris-citrate buffer containing 0 to 0.9 μM resiquimod (a TLR7/8 ligand) that affects only the X chromosome sperm. Sperm was then subjected to a swim-up test method. After incubation, sperm in the top and lower layers of the media were transferred into a separate new tube. Sperm motility characteristics, concentration, morphometry, and adenosine triphosphate (ATP) concentration from both layers were identified. The data were analyzed for multiple comparisons using one-way analysis of variance.

**Results:**

The study demonstrated that the presence of resiquimod used in the swim-up method facilitated the morphometric separation of smaller sperm, predominantly representing Y chromosome sperm in the top layer. Meanwhile, larger samples, indicating X chromosome sperm, accumulated in the lower layer of the media, thus increasing sperm concentration. Resiquimod also decreased motility in the lower layer, but samples in the top layer were unaffected. This decrease was reinforced by the depletion of the ATP level of sperm at the lower level.

**Conclusion:**

The TLR7/8 ligand reduced the motility of the putative X-sperm by decreasing their ATP content, allowing separation from the putative Y-sperm. These results suggested the importance of TLR7/8 as a potential biomarker in sperm selection technology.

## Introduction

Sperm separation is a type of assisted reproductive technology, comprising the precise separation of sperm containing X and Y chromosomes. This reproductive technology is beneficial for farmers seeking to improve economic returns through the strategic selection of animals based on sex. For example, dairy farmers prefer female animals to produce milk, while animal meat producers predominantly favor males. In addition, utilizing sexed sperm holds the potential to produce animals with improved genetic quality. Several studies have also shown that it is a feasible option to maintain the desirable sex ratio. The fundamental principle of sperm separation originates from the differences between X-bearing and Y-bearing sperm. Extensive studies have reported that X-bearing sperm has distinctive characteristics, including a higher DNA content, larger size, and slower motility compared to the Y-bearing variant.

In recent years, many sperm sexing techniques have been developed, but their commercial viability has been hampered due to their limited efficacy. However, the current technique has been successfully reviewed previously [1, 2]. Their investigations revealed that toll-like receptors 7/8 (TLR7/8), receptor proteins linked with the cell membrane, were exclusively present at the flagellum of the X-sperm but absent in the Y-sperm. The use of the TLR7/8 ligand was also reported to specifically reduce the X-sperm motility, thereby allowing for sperm sexing in mice [2]. The results obtained show new insights into the method of sperm sexing based on variations in sperm membrane-specific proteins [3] and a new reference for controlling livestock offspring. Despite these efforts, the impact of the TLR7/8 ligand is still unknown in sheep’s X-sperm and Y-sperm, particularly in quality.

According to previous studies, sheep are primarily raised for meat, milk, and fiber (wool) production. The demand for sheep meat, characterized by its high protein and fat content, has been reported to be substantial globally, and this demand is anticipated to increase due to population growth. Several reports have shown that the value of rams is significantly higher compared to ewe due to their higher daily weight gain and preference by the farmers [4]. In addition, the ratio of rams and ewes generated by natural mating is roughly 1 to 1, and the breeding of numerous undesired sex offspring typically increases production costs. This shows that there is a pressing need to develop various methods for sperm separation. Most X and Y chromosome-bearing sperm separation techniques in farm animals are based on differences in DNA content. Previous studies have also documented the successful use of TLR7/8 in separating sperm in mice [2], cattle [1, 5], and goats [6], but there are no reports on its usage in rams. Therefore, this study aims to determine the effect of the TLR7/8 ligand on inhibiting the movement of putative X-chromosome-bearing sperm in rams. The proportion of X- and Y-sperm based on morphometrical measurement in response to TLR7/8 ligand was also evaluated, along with its impact on sperm motility, concentration, and ATP concentration. The findings of this investigation are expected to provide new findings into the mechanisms underlying TLR7/8 ligand on the characteristics of sperm sexing and suggest its importance as a biomarker in a ram’s sperm sexing.

## Materials and Methods

### Reagents and animals

All chemicals, including tris (hydroxymethyl), citric acid, D-glucose, and resiquimod as a TLR7/8 ligand, were acquired from Sigma-Aldrich (St. Louis, CA). At the same time, the ATP bioluminescence assay kit II was obtained from Roche (Mannheim, Germany). Furthermore, semen was obtained from three fertile male rams utilizing an artificial vagina. The samples were then centrifuged for 5 min at 250x*g* with tris-citrate buffer (pH 7.2) to remove seminal plasma. The Universitas Padjadjaran Research Ethics Committee approved all animal research (approval no. 1420/UN6.KEP/EC/2023).

### Sperm incubation, morphometry, motility, and concentration

Sperm (50 × 10^6^) were incubated in 3 ml of tris-citrate buffer (332 mM tris, 83 mM citrate, and 22.2 mM glucose, pH 7.2) at 37°C with 0–0.9 μM resiquimod. Furthermore, the swim-up test was conducted using the method suggested by previous studies [2, 7]. After 60 min of incubation, the top layer (1 ml) was moved to a fresh tube, followed by 5 min of centrifugation at 250x*g*. The pellet was suspended in tris-citrate buffer, while the lower layer (1 ml) was shifted to a new tube, washed, and the pellet was suspended similarly to the top layer [5]. Furthermore, sperm morphometry from the top and lower layers was measured using DP20 software attached to a microscope (Olympus, IX71), and the concentrations were assessed using a Neubauer chamber.

Sperm motility of the top and lower layers was captured using video-microscopy for 5 sec and then classified into three groups; progressive, non-progressive, and non-motile [8]. In brief, progressive motile sperm had forwarded motion and slow motility, non-progressive motile vibrated in place, and non-motile had no movement, and the combination of progressive and non-progressive motile sperm represents the total motile.

### ATP quantification

The top and lower layer ATP concentrations were quantified using bioluminescence assay kit II (Roche, Manheim, Germany). Sperm was incubated with 0, 0.3, 0.6, or 0.9 μM resiquimod in a tris-citrate buffer containing 22.2 mM glucose for 60 min at 37°C. After swim-up incubation, sperm from the top and lower layers were incubated at 25°C for 5 min after being solubilized using a lysis reagent. Furthermore, after centrifuging at 10,000xg for 1 min, the supernatant from each layer was moved to a new tube and mixed with luciferase reagent*.* A multimode Tecan Infinite M200PRO plate reader was then used to measure the bioluminescence signal.

### Statistical analysis

One-way analysis of variance (ANOVA) was employed to compare multiple data sets, followed by Tukey’s honestly significant difference (HSD). The acquired data were presented as mean SEM, and a *p-value* of < 0.05 indicated that the differences were significant.

## Results

**Figure 1.**
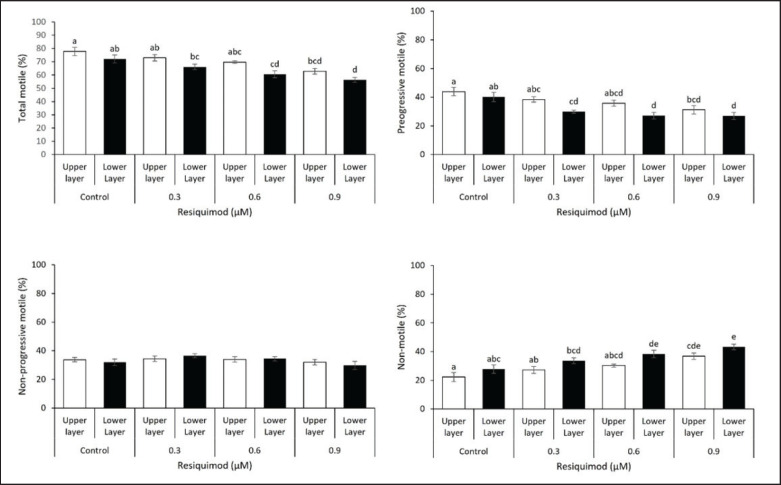
Motility characteristics of sperm in the top and lower layers in the presence of resiquimod supplementation. Spermatozoa were incubated with 0, 0.3, 0.6, or 0.9 μM resiquimod for 60 min at 37ºC. Total and progressive motile sperm in the top layer were reduced by the presence of 0.9 μM resiquimod compared to the control of the top layer. Dramatically decreased were observed in the presence of 0.6 and 0.9 μM resiquimod for total motile sperm, and 0.3, 0.6, and 0.9 resiquimod for progressive motile sperm compared to those in the control of lower layers, suggesting this TLR7/8 ligand suppresses the motility of X-containing sperm enriched in the lower layer. No differences were found in the non-progressive motile sperm in response to the presence of resiquimod. However, non-motile sperm were increased in the presence of resiquimod (0.9 μM in the top layer and 0.6 to 0.9 μM in the lower layer compared to those in each control). Data are expressed as the mean ± S.E.M. (*n* = 12). Different letters above columns indicate significant differences (*p < *0.05).

The impact of TLR7/8 ligand (resiquimod) was examined by incubating sperm in 0, 0.3, 0.6, or 0.9 μM resiquimod for 60 min at 37°C. After incubation, using a swim-up method, 1 ml of the top layer and 1 ml of the lower layer were separated and placed into new tubes. The findings presented in Figure 1 revealed that no variations were found in total motile (69.7%–77.7%), progressive motile (35.8%–43.9%), and non-motile (22.3%–0.3%) in the top layer media when the samples were incubated in 0–0.6 μM. However, total and progressively motile samples in the top layer were decreased in the existence of 0.9 μM in contrast to the top layer control (62.8% *vs.* 77.9% and 31.2% *vs.* 43.9%, respectively). A strong effect of resiquimod on sperm movements was observed in the lower layer. In the presence of 0.6 μM resiquimod, total motile (60.6%) and progressive motile (27.2%) were significantly lower compared to the lower layer control (total motile: 72.1% and progressive motile: 40.2%, respectively). A decrease in progressive motile sperm was also noted following the administration of 0.3 μM resiquimod. Furthermore, total motile and progressive motile decreased significantly with increasing concentration of resiquimod, suggesting the role of TLR7/8 ligand in sperm motility in the lower layer, which could be enriched by X-bearing-chromosome sperm. Resiquimod caused an increase in non-motile sperm in the top (0.9 μM: 36.8%) and lower layers (0.6 μM: 38.4%; 0.9 μM: 43.3%) compared to each control (the top layer: 22.3%; the lower layer: 27.8%). The results showed that there were no variations in the non-progressive motile sperm in either the top or the lower layer.

Considering morphometrical differences between X- and Y-sperm due to the quantity of DNA in the head, the head length, width, and area of sperm in both the top and lower layers were measured. The results revealed there were no differences statistically in the morphometrical measurements of head sperm in all groups (Table 1). However, the lower layer of media containing resiquimod tended to have larger head sperm dimensions (head length: 9.40–9.45 μm; head width: 5.23–5.30 μm; head area: 42.85^2^–43.28 μm^2^) compared to those in the top layer (head length: 9.14–9.39 μm; head width: 4.50–4.94 μm; head area: 38.99–40.63 μm^2^), showing distinction between X- and Y-sperm.

**Figure 2.**
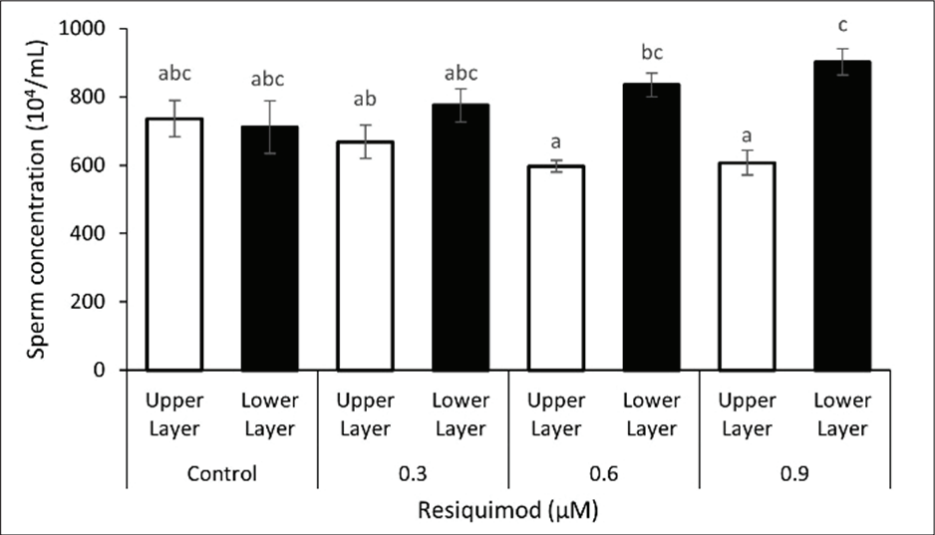
Sperm concentration generated from *swim-up* method containing different levels of resiquimod. Sperm concentrations in the top layers were lower than those in the lower layer when 0.6 or 0.9 μM resiquimod was present, indicating the X-sperm enriched in the lower layer due to the decrease of their motility. Data are expressed as mean ± SEM (*n *= 10). Different letters above columns indicate significant differences (*p < *0.05).

In line with the results presented in Figure 1, incubation with resiquimod increased sperm concentration in the lower layer (Figure 2). The presence of 0.9 μM resiquimod had the highest sperm concentration (9,02 × 10^4^/ml) and was significantly different from all the groups. Meanwhile, the presence of 0.6 μM resiquimod increased sperm concentration in the lower layer (8,35 × 10^4^/ml) compared to those in the top layer. The results showed no differences in the top layer with different concentrations of resiquimod. Based on these results, resiquimod accumulated X-sperm in the lower layer due to the decreased motility.

**Figure 3.**
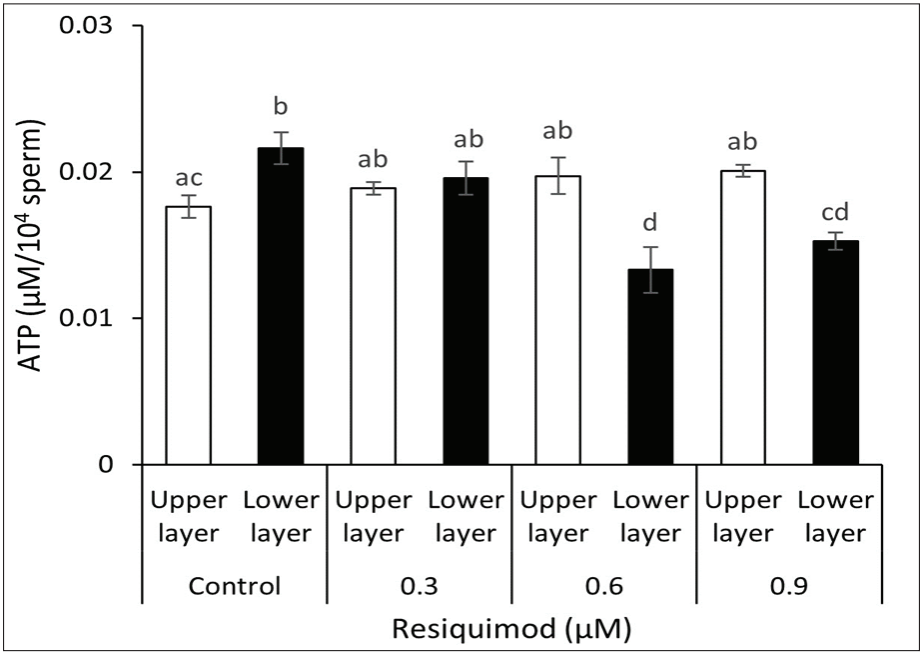
ATP concentration of sperm in response to different levels of resiquimod in the top and lower layers. The presence of 0.6 and 0.9 μM resiquimod significantly decreased ATP concentration of sperm in the lower layer of the media, indicating this TLR7/8 ligand suppresses ATP content of X-sperm that might enrich in the lower layer. Data are expressed as mean ± SEM (*n* = 4). Different letters above columns indicate significant differences (*p < *0.05).

**Table 1.** Measurement of sperm head after separation using different levels of resiquimod supplementation.

	Resiquimod (μM)							
	Control		0.3		0.6		0.9	
	Upper layer	Lower layer	Upper layer	Lower layer	Upper layer	Lower layer	Upper layer	Lower layer
Head								
Length (μm)	9.49 ± 0.03	9.34 ± 0.03	9.25 ± 0.02	9.42 ± 0.03	9.39 ± 0.03	9.45 ± 0.02	9.14 ± 0.03	9.40 ± 0.03
Width (μm)	5.25 ± 0.04	5.28 ± 0.03	4.90 ± 0.02	5.30 ± 0.03	4.50 ± 0.03	5.23 ± 0.02	4.94 ± 0.03	5.30 ± 0.03
Area (μm2)	43.31 ± 0.54	42.84 ± 0.36	39.10 ± 0.26	43.28 ± 0.37	40.63 ± 0.40	42.85 ± 0.29	38.99 ± 0.34	43.23 ± 0.38

Data are expressed as the mean ± SEM (*n* = 100 sperm). Different letters within rows indicate a significant difference (*p < *0.05).

To examine the roles of TLR7/8 in ram sperm ATP generation, the concentration of cellular ATP was measured in samples from different layers incubated with or without resiquimod. The results showed that there were no changes in the sperm ATP concentration among the top layer groups (0.017–0.020 μM) (Figure 3). However, when the concentration of resiquimod increased, the ATP concentration of sperm in the lower layer significantly dropped (0.6 μM resiquimod: 0.013 μM ATP; 0.9 μM: 0.015 μM ATP), which suggested TLR7/8 activation by resiquimod. These results proposed the involvement of TLR7/8 in ATP production in the X-sperm of rams.

## Discussion

Ram sperm conveyed either an X or Y chromosome, which was delivered along with autosomal chromosomes to the egg during fertilization at the oviduct. The fertilization of an egg by X- and Y-bearing-chromosome sperm typically led to the formation of female and male zygotes, respectively. A previous study had also shown that X- and Y-sperm existed in a ratio of 1:1 in an average ejaculate [9]. This showed that the ratios of female and male offspring generated by natural mating or regular artificial insemination were expected to be similar. Since the economic efficiency of the livestock industry was often related to livestock sex, the ability to produce predetermined offspring sex had the potential to increase profitability. Consequently, sperm sexing has been an exciting study topic in animal husbandry for decades [10]. In this current investigation, we discovered that sperm localized in the lower layer of media had lower motility characteristics and ATP production in the presence of TLR7/8 ligands compared to those in the top layer. Considering only X-bearing-chromosome sperm possessed TLR7/8, and resiquimod affected TLR7/8, the sample in the lower layer media was enriched with X-bearing-chromosome sperm.

Toll-like receptors are protein membranes of cell surfaces that mediate immune responses for various pathogen-derived ligands and link innate and adaptive immunity [11]. A previous study showed that TLR2/4 could disrupt fertilization by altering motility and capacitation when sperm or uterus were contaminated by bacteria or viruses [12]. A previous study also reported the participation of TLR signaling in sperm motility and mitochondrial activity through PI3K and GSK-3α in mice [13]. In this report, sperm motility decreased in the presence of resiquimod. Similar results were obtained in a previous study where resiquimod impaired sperm motility in mice, and its withdrawal led to restoration [2]. In addition, following the administration of resiquimod during a swim-up test, the sperm was divided into upper and lower sperm. The findings revealed significant changes in sperm motility in the lower layer, but not the upper layer, leading to an increased concentration of sperm in the lower layer (Figs. 1 and 2). Sperm sorting was distinguished between X/Y sperm using morphometrical measurement. Some studies had reported variations in the size of X- and Y-bearing sperm, where the heads of X-bearing variants were larger due to higher DNA content [14,15]. The results of this study demonstrated that there was no significant difference in size between the heads of X- and Y-sperm. Similar results were obtained from a previous study using photography captured with phase contrast microscopy [16]. Meanwhile, a previous report showed that the observed variance in head volume could be caused by changes in the DNA content of sperm harboring X and Y chromosomes [17,18]. The variation of DNA content between X- and Y-sperm ranged from 3.5% to –4% and was associated with differences in length, width, perimeter, and sperm head area. However, our findings revealed no variations in sperm head measurements in X- and Y-sperm, which was supported by the results obtained by Zavaczki *et al. *[16]. This was because the variation in DNA content was insufficient to be observed as a variation in the volume of sperm heads.

Sperm is characterized by their high motility, which requires a continuous ATP supply for cellular energy. In addition, ATP is generated by glycolysis and mitochondrial respiration, which is a source of energy for axonemal dynein ATPases in the flagellum to produce flagellar beat [19,20]. In this study, resiquimod decreased the level of ATP content of lower layer sperm, but not the upper layer sperm. Previous studies showed that ATP production accelerated the linear sperm velocity of various species [20–23], hence a decrease in ATP production could lead to low sperm motility. We observed that a TLR7/8 ligand reduced sperm motility by altering the ATP generation of lower sperm, leading to low fertility. This showed that further investigation regarding the restoration of lower layer sperm and their fertility rate is required.

## Conclusion

This report showed that resiquimod decreased the motility of putative X-sperm by decreasing ATP concentration, but did not affect putative Y-sperm. These results provided new insights into sperm separation methods in sheep to improve sheep production efficiency. As it was a preliminary study on the motility of sexed sperm, the study team had planned to conduct investigations to determine the successful rate of in vivo sheep production using TLR7/8-based sperm separation.

## List of Abbreviations

ANOVA, analysis of variance; ATP, adenosine triphosphate; DNA, deoxyribonucleic acid; GSK-3α, glycogen synthase kinase 3alpha; HSD, Tukey’s honestly significance difference; PI3K, phosphoinositide 3-kinase; SEM, standard error of the mean; TLR7/8, toll-like receptor 7/8.
